# Evaluation of a new semi-automated high-performance liquid chromatography method for glycosylated haemoglobins

**DOI:** 10.1155/S1463924686000378

**Published:** 1986

**Authors:** Assunta Carpinelli, Andrea Mosca, Pierangelo Bonini

**Affiliations:** 1 Universitá degli Studi di Milano Dipartimento di Scienze e Tecnologie Biomediche Via Olgettina 60 Milano I 20132 Italy; 2 Istituto Scientifico S. Raffaele Milano Italy


Journal of Automatic Chemistry/*Journal of Clinical Laboratory Automation*, Vol. 8, No. 4 (October–December 1986), pp. 192–196

### Evaluation of a new semi-automated high-performance liquid chromatography method for glycosylated haemoglobins

**Assunta Carpinelli, Andrea Mosca***

*Universitá degli Studi di Milano, Dipartimento di Scienze e Tecnologie Biomediche, Via Olgettina 60, I 20132 Milano, Italy*

**and Pierangelo Bonini**
*Istituto Scientifico S. Raffaele Milano, Italy*

### Introduction

The measurement of glycosylated haemoglobin in blood is vital for monitoring metabolic control in diabetics [1–3]. The development of many different methods of measurement (based on cation-exchange chromatography, electrophoresis, high performance liquid chromatography [HPLC], affinity chromatography, radio and colorimetric immuno techniques) proves the importance of this assay [4]. Since the first HPLC method was described [5] a number of modifications have been reported (see the review by Ellis *et al.* [6]). Instruments have been specially designed and commercialized (for example by Helena Laboratories, Beaumont, Texas, USA and Kyoto Daichi, Japan).

The evaluation of a recently commercialized semi-automatic HPLC instrument, which performs analyses on hemolysates from the original blood samples, is reported.

### Materials and methods

The instrument, commercialized by Bio-Rad Laboratories (Segrate, Milano, Italy), consists of a main unit, containing the functional parts (autosampler, buffer reservoir, pumps, column colorimeter, and a control unit). A single piston pump is used with a step-gradient valve system, by which the elution of the column is performed with three phosphate buffers of pH 5·7, 5·8 and 5·9 (unstated concentration) and increasing ionic strength. The analytical column (4·0 × 150 mm) is filled by a new cation exchanger (TSK gel, unstated composition). The column temperature is controlled at 23 °C by forced ventilation in the column compartment.

The sample (5 μl of venous blood, with 1 mg/ml of EDTA) is diluted automatically with 1 ml of haemolysing reagent (0·1% vol/vol polyoxyethylene-ether in borate buffer); 20 μl of the haemolysate are used for analysis. Spectrophotometric detection is performed at two wavelengths (415 nm and 690 nm) to ensure a stable base-line. All the operations are controlled by a microprocessor and a built-in integrator performs data reduction. The final results are printed out together with a chromatogram of the run. The order of elution of the haemoglobin fractions is: $HbA_{1a}$, $HbA_{1b}$, HbF, $HbA_{1c}$, $HbA_0$. Each run takes 8 min.

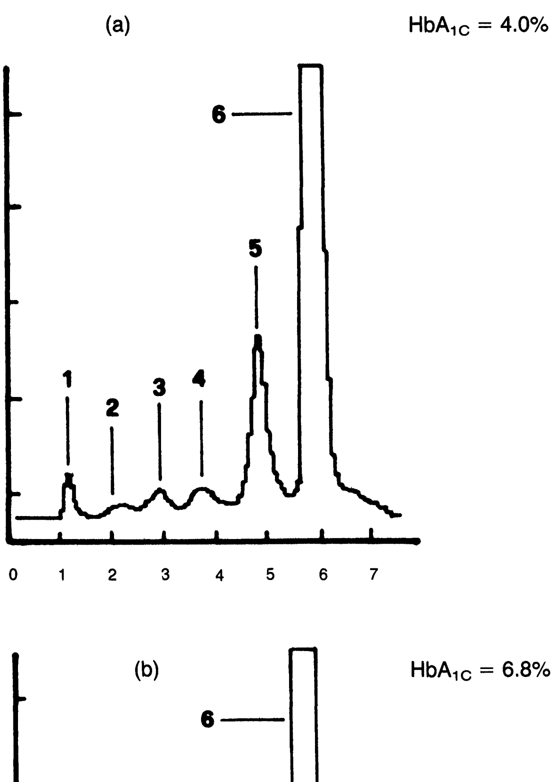

*Figure 1. Chromatograms of a normal (a) and a diabetic (b) sample obtained after a standard run. X-axis: time (min); y-axis: concentration, as a percentage of the total haemoglobin (upper scale: 10%). The fractions separated are: (1) non-haemoglobin peak; (2) $HbA_{1a}$; (3) $HbA_{1b}$; (4) HbF; (5) $HbA_{1c}$; and (6) $HbA_0$.*

* All correspondence to Dr Mosca.

The reference method used for the evaluation was the Bio-Rad Laboratories microchromatographic method for $HbA_{1c}$ performed at 23 °C, as previously described [7]. The alkali denaturation method, modified according to Molden [8], was used for HbF identification.

Blood samples used were collected by the Clinical Chemistry Laboratory and the Clinical Medicine Division (H. S. Raffaele, Milano, Italy). A Coulter S-Plus IV (Kontron, Switzerland) was used to measure the haematological parameters. Some ethylene glycol stabilized haemolysates were also prepared, according to a previously reported procedure [9], and used within two months.

Analytical imprecision was measured using blood samples and a lyophylised control material (Lypocheck Hemoglobin Alc, from Bio-Rad Laboratories).

### Results and discussion

Figure 1 shows the chromatograms obtained after the analysis of a normal (*a*) and a diabetic sample (*b*). The analyser separates four or five haemoglobin fractions depending on the HbF level (see later). The amount of each species and the order of elution are closely similar to those obtained by liquid chromatography with the Bio-Rex 70, under the conditions reported by Wacks *et al.* [10]. In order to test whether or not the amount of haemoglobin loaded to the column would interfere with analytical accuracy, as reported for the microchromatographic methods [11], samples with different haematocrit values and similar glycosylated haemoglobin levels were analysed. The samples were prepared by diluting packed red blood cells with different volumes of their own plasma. The results obtained showed that, for haematocrits between 22% and 75%, no appreciable effect is produced on the results of analysis, i.e. no haematocrit-effect can be detected.

The analytical precision, within and between runs, was also analysed; the results obtained are reported in table 1.

In order to evaluate the within-series precision the analyses were repeated eight times on a blood sample from a normal subject, 10 times on blood from a diabetic patient and 17 times on the control lyophylised material.

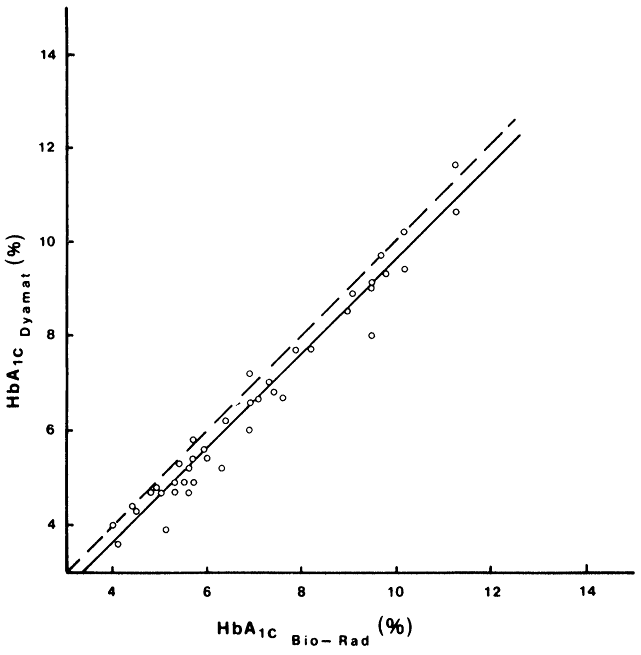

*Figure 2. Comparison with the reference method in the $HbA_{1c}$ determination. The equation derived from the linear regression analysis is: $y = 0{\cdot}991 \times -0{\cdot}32$; $n = 43$; $r = 0{\cdot}985$, $P < 0{\cdot}001$. The dashed line correspond to the theoretical regression equation: $y = 1{\cdot}000 \times +0{\cdot}00$.*

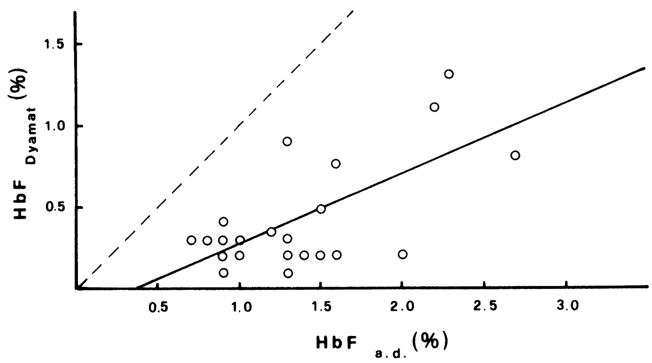

*Figure 3. Comparison between the present method and the alkali denaturation method, for the measurement of HbF ($y = 0{\cdot}41 \times -0{\cdot}12$; $n = 29$; $r = 0{\cdot}634$; $P < 0{\cdot}001$). Dashed line as in figure 2.*

*Table 1. Precision. For each set of data the mean and the standard deviation (as a percentage of the total haemoglobin) are reported. Coefficients of variation are shown in parentheses.*

| Sample | n | $HbA_{1a}$ | $HbA_{1b}$ | HbF | $HbA_{1c}$ |
|---|---|---|---|---|---|
| *Within-run* | | | | | |
| Normal | 8 | 0·3 ± 0·1 (0·0) | 0·5 ± 0·1 (10·0) | 0·5 ± 0·1 (7·2) | 4·1 ± 0·1 (1·1) |
| Diabetic | 10 | 0·4 ± 0·1 (13·0) | 0·6 ± 0·1 (5·2) | — | 6·8 ± 0·1 (1·0) |
| Lyphocheck $A_{1c}$ | 17 | 1·1 ± 0·1 (4·2) | 1·9 ± 0·1 (3·0) | — | 7·3 ± 0·1 (1·5) |
| *Between-run* | | | | | |
| Low-level ethylene glycol haemolysate | 9 | 0·3 ± 0·1 (13·7) | 0·6 ± 0·0 (0·0) | — | 4·6 ± 0·1 (2·5) |
| High-level ethylene glycol haemolysate | 9 | 0·3 ± 0·1 (8·1) | 0·6 ± ·1 (4·0) | — | 7·3 ± 0·1 (1·4) |

| NAME | % | TIME | AREA |
|---|---|---|---|
| A1A | 0.4 | 2.3 | 34.92 |
| A1B | 0.7 | 2.9 | 68.05 |
| A1C | 5.1 | 4.8 | 512.07 |
| A0 | 93.8 | 5.7 | 9355.29 |

HBA1C 5.1% HBA1 6.2%

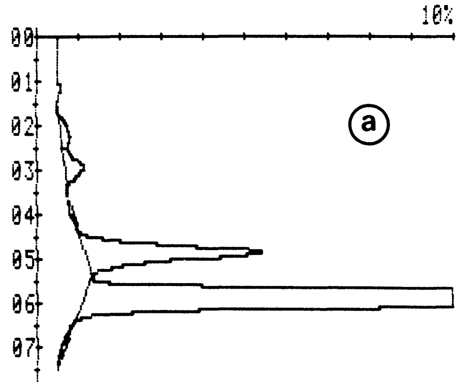

| NAME | % | TIME | AREA |
|---|---|---|---|
| A1AB | 0.1 | 2.3 | 8.72 |
| F | 0.5 | 2.9 | 78.83 |
| A1C | 0.3 | 3.9 | 46.37 |
| A0 | 2.5 | 4.8 | 420.53 |
| P5 | 56.1 | 5.7 | 9550.11 |
| P6 | 40.7 | 6.1 | 6923.71 |

HBA1C 0.3% HBA1 0.3%

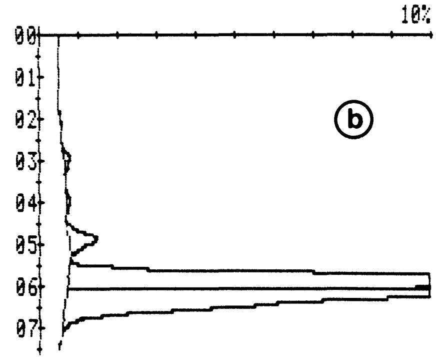

| NAME | % | TIME | AREA |
|---|---|---|---|
| A1A | 0.1 | 2.3 | 13.34 |
| A1B | 1.0 | 2.9 | 121.65 |
| A1C | 1.2 | 3.8 | 143.68 |
| A0 | 4.7 | 4.8 | 560.07 |
| P5 | 92.9 | 5.7 | 11004.71 |

HBA1C 1.2% HBA1 2.4%

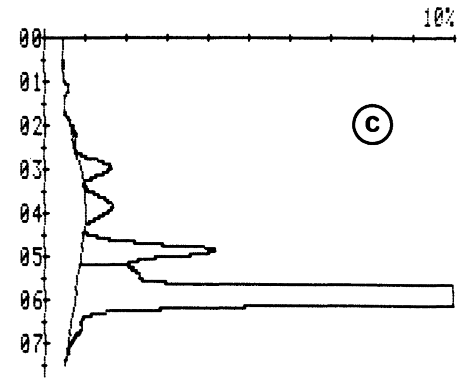

| NAME | % | TIME | AREA |
|---|---|---|---|
| A1AB | 13.1 | 2.5 | 1032.81 |
| A1C | 0.7 | 4.2 | 53.44 |
| A0 | 4.4 | 4.9 | 345.36 |
| P4 | 81.8 | 5.8 | 6453.16 |

HBA1C 0.7% HBA1 13.8%

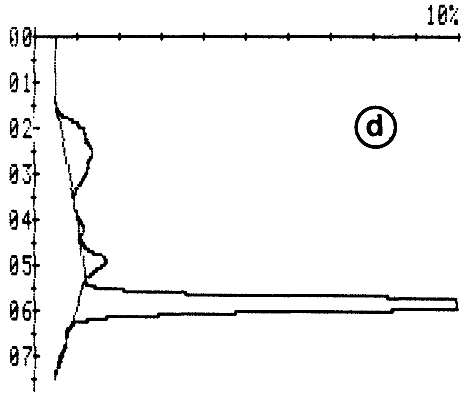

| NAME | % | TIME | AREA |
|---|---|---|---|
| A1AB | 0.3 | 2.3 | 27.67 |
| F | 3.1 | 2.9 | 301.63 |
| A1C | 2.5 | 3.9 | 247.49 |
| A0 | 3.2 | 4.8 | 313.86 |
| P5 | 91.0 | 5.7 | 8987.27 |

HBA1C 2.5% HBA1 2.8%

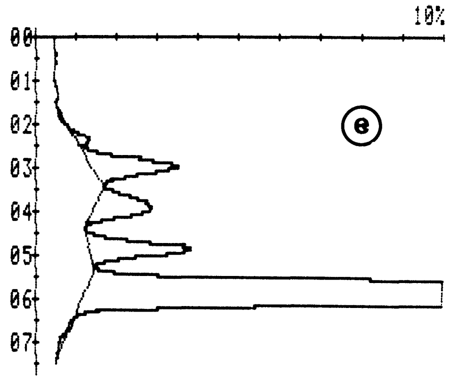

*Figure 4. Chromatograms obtained from the analysis of haemolysates from subjects suffering from different haemoglobinopathies: (a) normal control; (b) HbS (β6 glu → val) trait; (c) HbE (β26 glu → lys) trait; (d) HbJ Paris (α12 ala → asp) trait; and (e) Hb Lepore trait (δβ → fusion). The concentrations of mutant haemoglobins in each sample, determined by cellulose acetate electrophoresis at pH 8·4, with respect to the total haemoglobin concentration were: HbS = 38%; HbE = 33%; HbJ Paris = 24%; and Hb Lepore = 12%.*

The between-run precision was tested over a period of one month, analysing two ethylene glycol stabilized haemolysates nine times (every third day). Each daily measurement was performed in duplicate.

The accuracy in the measurement of $HbA_{1c}$ was evaluated by measuring this fraction in 41 blood samples with both the present method and the reference method. The data obtained are shown in figure 2.

The accuracy of HbF measurement was determined on a limited number of samples, the data being reported in figure 3. It can be seen that the accuracy is not satisfactory. Further methodological improvements must be introduced to resolve this quantitatively minor fraction which has great clinical relevance for the characterization of the β-thalassemia syndromes.

Interference by abnormal haemoglobins was investigated by analysing haemolysates from subjects suffering from various haemoglobinopathies, which had previously been characterized. Figure 4 shows that in all the specimens analysed, the instrument produced an abnormal chromatogram; if an abnormal haemoglobin was present it showed as an anomalous peak, indicated as P4, P5 or P6.

In the case with HbS (see figure 4[*b*]), the $HbA_0$ peak is split into two fractions, the last one (P6) probably corresponding to the HbS present in the sample. There is good correlation between the amount of this fraction (40·7%) and the HbS concentration in the sample (38%) determined separately by cellulose acetate electrophoresis (CAE) at pH 8·4. The position of the peak suggests also that the fraction may have an electrophoretic mobility slower than that of $HbA_0$, as is to be expected for HbS.

A somewhat similar comparison holds for the sample showing the HbJ Paris trait (see figure 4[*d*]). This haemoglobin variant has greater mobility than $HbA_0$ on CAE, and it is therefore expected to be eluted among the first fractions. The chromatogram shows an elevation in the $HbA_{1a}$ and $HbA_{1b}$ fractions, which are eluted together. The concentration of these fractions (13·1%) is too high even for a sample from a diabetic person, and indicates the presence of interference. The concentration found using this analysis is significantly lower than that measured by CAE.

In the case of the Hb Lepore trait (see figure 4[*e*]), a variant with electrophoretic mobility close to that of HbF, an abnormal chromatographic pattern is shown, but detection of the type of the variant from the chromatogram is very uncertain.

Characterization is also impossible in the case of the sample showing the HbE trait (see figure 4[*c*]). This mutant has a very low electrophoretic mobility at pH 8·4 (similar to that of $HbA_2$) and elution is therefore expected after $HbA_0$. In this case the $HbA_0$ peak is not split, as with the HbS trait sample, but the chromatogram is abnormal at the boundary between the $HbA_0$ peak (elution time = 5·3 min). The final peak shows a small shoulder on its right-hand side.

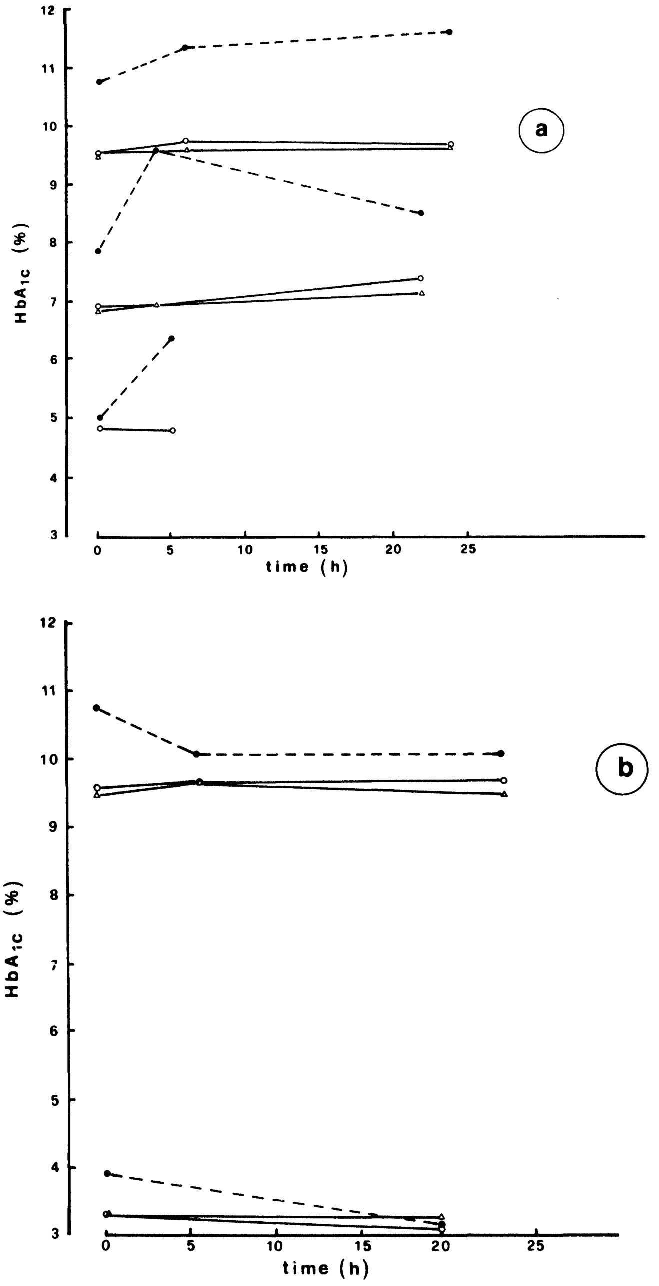

*Figure 5. The effect of incubation with 4·0 g/l glucose (a) and 9.0 g/l NaCl (b) on the assay of glycosylated haemoglobins. Incubations were performed at 37 °C using erythrocytes from two normal and two diabetic subjects. The reagents used were: (○——○) Glycopack haemolysing reagent 30′ at room temperature; (△——△) Glycopack haemolysing reagent 30′ at 37 °C; (● – – ●) haemolysing reagent without borate buffer, 10′ at 25 °C.*

From our experience we can therefore conclude that the instrument is more sensitive to the presence of abnormal haemoglobins in the sample than are the minicolumns which are normally used. The operator should be alerted by the appearance of anomalous peaks but further electrophoretic investigations need to be performed from full diagnosis. The estimation of the true $HbA_{1c}$ levels can be attempted by a careful observation of the chromatogram, knowing that, under standard conditions, the elution time for $HbA_{1c}$ is about 4·8 min.

The influence of so-called 'labile glycosylated haemoglobin' fractions on this method was also examined. Erythrocytes were incubated separately with glucose (4·0 g/l) and with saline solution (9·0 g/l NaCl) at 37 °C, for up to 24 h. No significant increase in the $HbA_{1c}$ levels of three blood samples (one from normal and two from diabetic subjects) after 24 h of incubation with the glucose solution was detected (see figure 5[*a*]). Similarly, no significant decrease in the initial $HbA_{1c}$ levels was found in two blood samples (one normal and one diabetic) incubated with saline for 24 h (see figure 5[*b*]). Furthermore, in order to evaluate the presence of the labile fractions in the incubated erythrocytes, the same samples were analysed for $HbA_{1c}$ levels, using an haemolysing reagent without borate (the same haemolysing reagent previously commercialized Bio-Rad Laboratories for assays of $HbA_1$). This kind of reagent does not eliminate the aldimine forms during the haemolysis step. In fact, the results of these determinations (see the dashed lines in figure 5) showed an increase in the $HbA_{1c}$ levels after the glucose incubations, and a significant decrease in the $HbA_{1c}$ concentrations after the incubations with saline.

It can therefore be concluded that the method described here is insensitive to the presence of the 'labile fractions', which are reported to interfere strongly with many chromatographic methods.

In this evaluation no experiment was performed to test the effect of temperature on the analytical resolution of the minor haemoglobin fractions. Reports in the literature indicate that this effect is evident with all the procedures based on ion-exchange chromatography and, to a smaller extent, with those using affinity chromatography [12]. It is assumed that a thermoregulated column compartment within the apparatus will eliminate any interference from temperature variations on the chromatographic pattern.

In summary, it would appear that this system could be of significant value for the measurement of glycosylated haemoglobin. It combines good precision and accuracy with practicality for speed, technical skill requirements and safety. The system can also be useful in evaluating samples from subjects suffering from some haemoglobinopathies where diagnosis may frequently be missed by conventional chromatographic techniques, normally used to measure glycosylated haemoglobin levels.

### References

1. Goldstein, D. E. *New England Journal of Medicine,* **310** (1984), 384.

2. Miedema, K. K. and Casparie, T., *Annals Clinical Biochemistry,* **21** (1984), 2.

3. Ladenson, J. H., Kwok-Ming, C. and Kilzer, P., *Clinical Chemistry,* **31** (1985), 1060.

4. Goldstein, D. E., Wiedmeyer, H. M., England, J. D., Little, R. R. and Parker, K. M., *Critical Reviews of Clinical Laboratory Sciences,* **21** (1984), 187.

5. Cole, R. A., Soeldner, J. S. and Bunn, H. F., *Metabolism,* **27** (1978), 289.

6. Ellis, G., Diamandis, E. P., Giesbrecht, E. E., Daneman, D. and Allen, L. C., *Clinical Chemistry,* **30** (1984), 1746.

7. Carenini, A., Mosca, A. and Pozza, G., *Clinical Chemistry,* **29** (1983), 1687.

8. Molden, D. P., Alexander, N. M. and Neely, W. E., *American Journal of Clinical Pathology,* **77** (1982), 568.

9. Mosca, A., Carpinelli, A., Paleari, R., Bonini, P. A. and Franzini, C., *Journal of Clinical Chemistry and Clinical Biochemistry,* **23** (1985), 361

10. Wacks, M. T., Starkman, H. S. and Soeldner, J. S., *Clinical Liquid Chromatography,* Vol. II (CRC Press, Boca Raton, Florida, 1984), 131.

11. Mosca, A., Carenini, A., Samaja, M. and Saibene, V., *Journal of Laboratory Medicine,* **8** (1981), 401.

12. Kortland, W., van Rijn, H. J. M., Hoeke, J. O. O. and Thijssen, J. H. H., *Annals of Clinical Biochemistry,* **22** (1985), 261.

### SEMINAR

*22 and 23 October 1986: Minnetonka, Minnesota, USA*

The Seminar, entitled 'Crossflow Membrane Filtration', will be oriented toward engineers, managers or persons responsible for water treatment, pollution control or process engineering. Topics covered include the use of cross-flow RO, UF and MF membranes for industrial water treatment, medical/pharmaceutical water treatment, waste water reclamation and the recovery of metal salts, oils and other organics from waste or process streams. The seminar will cover the fundamentals of reverse osmosis, ultrafiltration and microfiltration, total system design considerations, storage and distribution of water, and the 'zero discharge' approach to pollution control. Hands-on equipment operation, installation, start-up and repair is included.

*For more information contact Laura Wedding, OSMO Membrane Systems Division, OSMONICS, INC. 5951 Clearwater Drive, Minnetonka, MN 55343, USA. Tel.: 612 933 2277.*

